# Rescue, Rehabilitation, and Feeding Schedule of a Franciscana Dolphin (*Pontoporia blainvillei*) Calf Stranded in Argentina

**DOI:** 10.3390/ani14192762

**Published:** 2024-09-24

**Authors:** Julio Daniel Loureiro, Juan Pablo Loureiro, Sergio Rodriguez Heredia, Lorenzo von Fersen, Karina Cecilia Alvarez, María Julieta Olocco Diz, Romina Nuñez Favre

**Affiliations:** 1 Oceanario Mundo Marino, San Clemente del Tuyú B7105, Argentina; loureirojd@gmail.com (J.D.L.); juanploureiro@gmail.com (J.P.L.); 2 Fundación Mundo Marino, San Clemente del Tuyú B7105, Argentina; pappokari@hotmail.com (S.R.H.); cecikaru@hotmail.com (K.C.A.); 3 Facultad de Ciencias Veterinarias, Universidad Nacional de La Plata, La Plata B1900, Argentina; 4 Nuremberg Zoo & YAQU PACHA, 90480 Nuremberg, Germany; lorenzo@vonfersen.org; 5 Facultad de Ciencias Veterinarias, Universidad Nacional de Buenos Aires, Ciudad Autónoma de Buenos Aires C1427CWO, Argentina; jolocco@fvet.uba.ar; 6 Consejo Nacional de Investigaciones Científicas y Tecnológicas, Ciudad Autónoma de Buenos Aires C1425FQB, Argentina

**Keywords:** nutritional profile, feeding formula, daily weight gain, hematology, healthcare protocols

## Abstract

**Simple Summary:**

The franciscana dolphin (*Pontoporia blainvillei*) is a small cetacean that is endemic to the coastal waters of the southwestern Atlantic Ocean, ranging from Espirito Santo (Brazil) to the northern coast of San Matías Gulf (Argentina). The franciscana lives in shallow waters, less than 50 m deep, and is subject to high levels of bycatch mortality in gillnets due to commercial and artisanal fishing activities. Consequently, it has been considered a vulnerable species by the IUCN (International Union for the Conservation of Nature) since 2006. The objective of this study is to present the case of the rehabilitation of a franciscana dolphin calf that was stranded in San Clemente del Tuyú, Argentina, at the beginning of the predation period. This study describes the husbandry conditions and feeding schedule used during the rehabilitation until its death, 88 days after admission. The results of hematological, cytological, and fecal analyses conducted during the rehabilitation period, as well as its diet, which was prepared by hand, are presented. These findings provide an important baseline for future studies on franciscana dolphins. This information may also be useful in the implementation and development of healthcare protocols for this species.

**Abstract:**

The franciscana dolphin (*Pontoporia blainvillei*) is a small cetacean endemic to the coastal waters of the southwestern Atlantic Ocean. Due to its restricted distribution, it is subject to high bycatch mortality in the gillnets used for commercial and artisanal fishing. The rehabilitation of the franciscana is still a major challenge, as most attempts to rehabilitate stranded animals have failed. This study aims to present the case of the rehabilitation of a franciscana dolphin calf, stranded in San Clemente del Tuyú, Argentina, at the beginning of the predation period. The feeding strategy and nutritional profile at different stages during the 88 days of rehabilitation are meticulously detailed. Its diet was prepared by hand based on studies of milk composition, the feeding ecology of franciscana in Argentinean waters, and previous records of the Fundación Mundo Marino Rehabilitation Center. The diets were designed to meet the nutritional needs of franciscana dolphins. In addition, the hematological, cytological, and fecal analyses recorded during the rehabilitation are presented. Although the animal could not be released, due to its death, this report provides baseline information that can improve the ability of veterinarians to care for debilitated, live-stranded dolphins. This information may also be useful in the implementation and development of healthcare protocols for this species.

## 1. Introduction

Franciscana dolphins (*Pontoporia blainvillei*; Gervais & d’Orbigny, 1844) are endemic to the coastal waters of the southwestern Atlantic Ocean. It is discontinuously distributed from Espirito Santo (18°25′ S, Brazil) to the northern coast of San Matías Gulf (41°10′ S, Argentina) [1]. The franciscana lives in shallow waters, less than 50 m deep, with the greatest density between the coast and 30 m depths and enclosed bays or estuaries [2]. This coastal distribution has resulted in high levels of bycatch mortality in gillnets due to commercial and artisanal fishing activities [1]. As a consequence, this species has been listed as “Vulnerable” by the IUCN Red List [3] and is also listed as “Vulnerable” on the Red List of Mammals of Argentina [4].

From 1987 to 2012, the Fundación Mundo Marino Rehabilitation Center (FMMRC) recorded 112 franciscana dolphin strandings (38 live and 74 dead). These animals belonged to three different categories: 57 corresponded to adult animals (dolphins that have reached sexual maturity; they are fully grown and able to reproduce), 42 were calves at the weaning period (young dolphins that are still nursing but are beginning to transition to solid food) [5], and 13 were juveniles (dolphins that are weaned from their mother’s milk but have not yet reached full sexual maturity). Because some of the carcasses were badly decomposed or only body parts were found, the gender of only 70 animals was identified, 36 females and 34 males. The distribution of stranded franciscana dolphins over the years shows that 93% of the strandings occurred between November and April (late spring, summer, and early fall). In terms of the animal category, calf dolphins at the weaning period were found stranded between November and February (with one recorded in October); juvenile strandings occurred between November and February, with one recorded in October and one in June; and adult strandings occurred throughout the year, with a peak between January and April (*n* = 50).

The rehabilitation of stranded franciscana dolphins remains a major challenge, as most attempts to rehabilitate stranded animals have failed [6]. Our records indicate that 38 franciscana dolphins were life-stranded and transported to the FMMRC. Of these, 15 animals (40%) died within the first 24 h, with 14% of these deaths occurring in the first 4 h. Furthermore, 19 animals (50%) died within the first week, while two animals (5%) died within one month. One animal survived for 88 days (current case), and another survived for almost 40 months [7].

Studies on the diet of franciscana have shown that it is an opportunistic feeder, ingesting the most frequent prey in the area (juvenile fish and squid). This behavior can be observed in the seasonal fluctuations in its diet, which coincide with the pattern of variation observed in the abundance of prey species throughout the year [8]. In northern Argentina, *Pontoporia blainvillei* consumes approximately 26 prey species, including teleost, crustaceans, and cephalopods [8]. However, at the beginning of the predation period (about 78–98 cm in length and 8–13 kg), eleven prey species were identified, including the striped weakfish (*Cynoscion guatucupa*); the long-finned squid (*Loligo sanpaulensis*); and the lesser important white croakers (*Micropogonias furnieri*), banded croakers (*Paralonchurus brasiliensis*), and crustaceans [5].

The rehabilitation of stranded cetaceans, particularly endangered species, is crucial for several reasons. First, each rehabilitation effort provides invaluable insights into the biology, behavior, and health of these animals. Detailed observations and medical interventions during the rehabilitation process provide data that are otherwise difficult to obtain from wild populations. This knowledge can help to refine conservation strategies, improve medical treatments, and increase our understanding of cetacean physiology and pathology. Secondly, by successfully rehabilitating and returning stranded whales to their natural habitats, we are directly contributing to the recovery of endangered species. This is particularly important, as each individual contributes to the genetic diversity and overall resilience of these vulnerable populations. In addition, rehabilitation efforts often increase public engagement and awareness, leading to greater support for marine conservation initiatives.

Even if the rehabilitation of stranded animals arouses increasing interest, the chances of being successful are in need of improvement. A review of 25 years (1977 to 2002) of data on live-stranded odontocetes (*n* = 70) from northern California revealed that their survival rates are relatively low [9]. Thirty-five animals (50%) died within the first 24 h of rescue, thirteen animals (19%) died within the first week, seven animals (10%) died within one month, and five animals (7%) survived more than one month but subsequently died. Three animals (4%) were deemed unreleasable and were returned to captivity, while five animals (7%) were released back into the wild. The overall effectiveness of rehabilitation remains low, and there is considerable debate about its benefits and drawbacks. According to Moore et al. (2007), opinions on the rehabilitation of stranded marine mammals are divided. Initially, rehabilitation was associated with display collections, but now it is primarily aimed at release. The reasons for rehabilitation include animal welfare, conflict management, research, conservation, and public education. However, these must be weighed against the costs and risks, such as resource conflicts, pathogen introduction, and concerns about long-term survival [10].

This study aimed to present the case of the rehabilitation of a franciscana dolphin calf at the beginning of the predation period stranded in San Clemente del Tuyú, Argentina.

## 2. Materials and Methods

**Figure 1:**
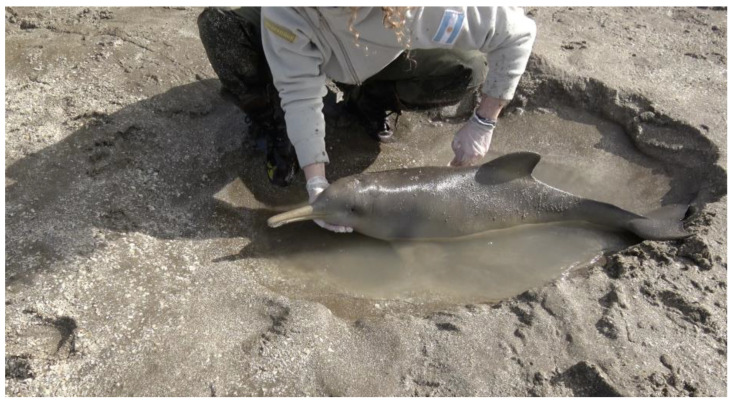
Initial care of *Pontoporia blainvillei* dolphin.

This study was conducted on a franciscana dolphin (*Pontoporia blainvillei;* Gervais & d’Orbigny, 1844) calf stranded on the coast of San Clemente del Tuyú (36°25′ S, 56°41′ W) in the summer of 2012 (23 January). The rescue crew performed an in-place physical examination and provided first aid attention. The initial care was oriented to keep the animal comfortable and minimize stress, which included overheating protection by placing its flippers and flukes in contact with water in their natural position, protecting its eyes and blowhole from sand, and skin protection from sunburn and rough surfaces (Figure 1).

**Figure 2:**
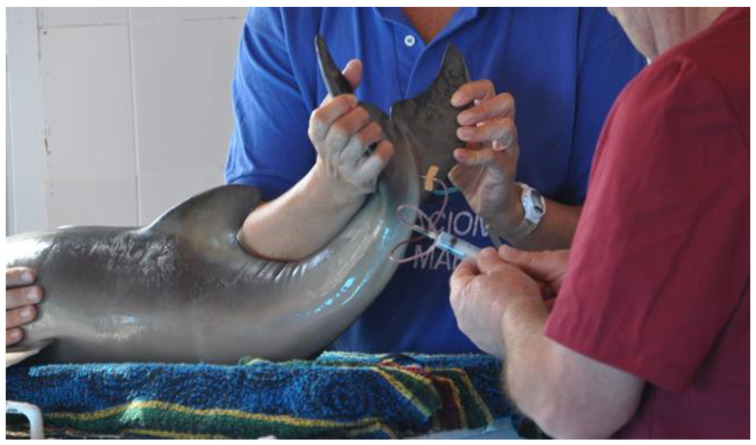
Blood sample collection.

This initial evaluation indicated that the calf was injured, and the mother was not visualized, so it was transported to the FMMRC. The dolphin was placed on a stretcher and transported in a pickup box covered with foam pads and mattresses, with wet towels placed on the animal’s body to keep the skin moist and prevent lesions. Once at the FMMRC, a complete medical examination was performed, and several samples were collected. Blood samples were collected from the coccygeal vein of the caudal peduncle [6,11] with a 25G × 3/4-inch needle butterfly and a 3 cc syringe (Figure 2). The coccygeal vein was selected as the sampling site due to its greater accessibility than the vein of the caudal peduncle in small cetaceans. To obtain a sputum sample for bacteriological analysis, the blowhole was first cleaned to remove water and other contaminants. Following one expiration, the blowhole was flushed with sterile saline. A second expiration resulted in removing most of the saline solution, after which the blowhole was dried with a sterile absorbent swab to eliminate any residual material. A sterile, non-absorbable collection vial was then placed over the blowhole and kept there until exhalation or “chuff” was collected in the vial [12]. Different culture media (EMB, Blood Agar, and Sabouraud Agar) were used for the bacteriology and mycology cultures. A sterile swab was gently inserted 5 cm into the rectum to perform the fecal bacteriological analysis. A stool sample was collected with a probe for a cytological and parasitological evaluation of the feces material [12].

After the initial evaluation, it was placed in a canvas circular pool, 90 cm in depth with a total volume of 10.000 L, filled with natural saltwater (salinity 23.62 ppt, average salinity of the estuary), with regular chlorination levels (total 0.38 ppm, free 0.20 ppm, and combined 0.18 ppm) and a pH around 7.7. The water was filtered continuously using sand filters, and warm water was added to maintain the temperature at around 23 °C. The bacterial coliform count was monitored once per week and maintained under 100 MPN in 100 mL water according to national regulations (Ley Provincial de Pesca Nº 11.477 and Decreto Reglamentario 3237/95 section IV, art. 13° inc. h) [13].

Its respiratory frequency was closely supervised (eight times a day) until the end of this study. To this end, an observer was situated at a distance that did not disrupt the animal’s behavior, and the number of expirations occurring within a 10 min period was recorded [6,14]. The body weight was monitored daily. Its body temperature was determined using a rectal thermometer placed 5 cm into the rectum [15]. During the rehabilitation period (88 days), a clinical evaluation, including behavioral records, physical evaluation, vital signs, and biological samples, were collected according to the requirements of the animal.

Since most stranded animals tend to be dehydrated, the initial treatment was based on rehydration with a diluted oral electrolyte solution until the blood electrolyte concentration results were obtained [16]. Once rehydration was achieved, nutrition was the major challenge during the next period.

### Feeding Protocol

The current franciscana dolphin was at the beginning of the predation/weaning period. Thus, the feeding regime was oriented to start with a milk replacer formula, followed by a transitional change to solid food. The animal’s weight was monitored daily to adjust the caloric intake. The feed formula was based on artificial milk formulas for cetaceans [17], studies of the milk composition of franciscana dolphins [18], the diet composition of franciscana in Argentinean waters [8], and previous records from the FMMRC. The nutritional profile of the different formulas used can be seen in Table 1. Each formula was prepared fresh daily and stored in a refrigerator until used. The formula was warmed to body temperature before feeding, and any remanent was discarded after 24 h.

The milk replacer was prepared with blended shrimp (*Artemisia longinaris*), Saracca loin (*Brevoortia aurea*), King weakfish (*Macrodon ancylodon*), fish oil (1g, Omega 3, Natufarma, Argentina), Dextrose 100% (Nutrosa, Nutricia Bago, Argentina), and hydration salts (Electrolitos Plus, Ruminal, Argentina). This formula was used for one week and supplied 675 Kcal/day (about 68.33 Kcal/kg). Based on feeding acceptance, during the second week, the food intake was increased to 1150 Kcal/day (about 114.16 Kcal/kg), and solid food, using Golden croaker (*Micropogonias furnieri*) or King weakfish (*Macrodon ancylodon*; without head and viscera), was incorporated. A proximate analysis of the feed ingredients was used to calculate the kilocaloric intake using the Atwater system. For this purpose, a whole golden croaker fish (*Micropogonias furnieri*) and a King weakfish without the head and viscera (*Macrodon ancylodon*) were used.

From weeks three to nine, the blended formulation was gradually replaced by solid food, and the nutritional intake was about 1750 Kcal/day (approximately 119 Kcal/kg).

After week nine, the diet schema supplied about 1300 Kcal daily, approximately 96 Kcal/kg, and was almost entirely composed of solid food. Additionally, a multivitamin complex was added to the formulations (Supradyn^®^, Bayer, Argentina; 1 comp, PO, SID). The details of the formula preparations are shown in Table 2.

## 3. Results

### 3.1. Medical Examination and Treatment

**Figure 3:**
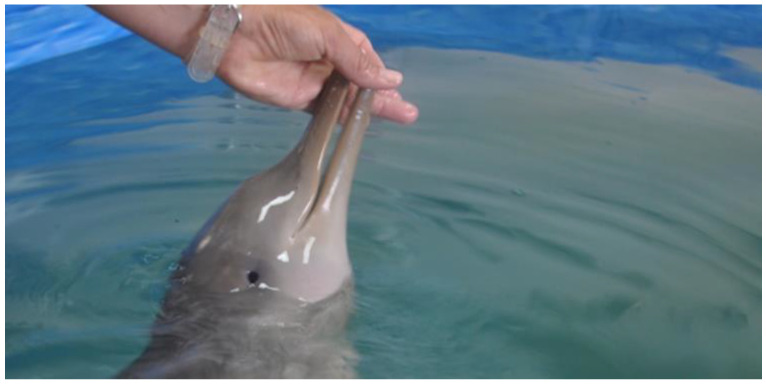
The superficial epithelial wound between the eye and the blowhole on the *Pontoporia blainvillei* calf rehabilitated at the FMMRC.

The medical examination revealed that the franciscana dolphin was a female with a length of 96 cm and a weight of 9 kg at the beginning of the predation/weaning period [5]. It was alert and responsive, with a body temperature of 36.6 °C and a respiratory rate of 42 breaths in 10 min. It presented with muffled lung sounds on auscultation and superficial epithelial wounds on the rostral right lateral part of the head (between the eye and the blowhole; Figure 3) and on both caudal fins.

Immediate supportive care was initiated during the first 24 h, focused on rehydration. A hydration solution of equal parts lactated Ringer’s and hydration salts (Electrolitos Plus, Ruminal, Argentina) in water was administered slowly through a gastric tube every 2 h. Approximately 60–70 mL was administered each time.

**Figure 4:**
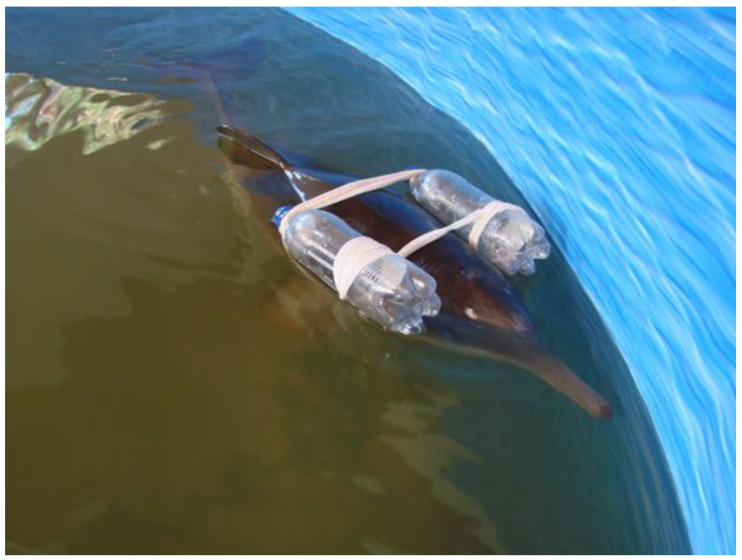
Calf assisted with flotation device to facilitate breathing and reduce swimming effort.

An abnormal swimming pattern was observed, with unilateral buoyancy and difficulty breathing (fast and superficial). Ultrasonography was performed using a Mindray DP30 with a 4.5 MHz probe. A confluence of pulmonary B-lines was observed in the projection area of the right and left cranial lobes, resulting in multiple areas of subpleural consolidation and one area of pulmonary consolidation. As this may indicate pulmonary edema due to saltwater inhalation during stranding, it was immediately treated with a unique dose of furosemide (1 mg/kg, IM, SID) and enrofloxacin (5 mg/kg IM, BID) for 10 days. Additionally, it was assisted with a flotation device with two floats to facilitate breathing and reduce its swimming effort (Figure 4). The flotation device was used until the animal was able to remain stable, which was after one week. Then, the device was removed. Its respiratory frequency showed an average rate of 45 breaths in 10 min during the rehabilitation (4–5/min). The respiratory fluid analysis was not conclusive due to the scarcity of the sample material.

Additional medications included in the food were anti-gas Factor AG (Simeticona 40 mg/mL, Laboratorio Casasco, Argentina; 15 drops BID) and lactobacillus (acidophilus; 1 drop six to nine times per day). As secondary fungal infections are common in calves receiving antibiotic treatment, itraconazole (2.5 mg/kg; BID) was added for 10 days beginning in week three [16].

Blood samples were collected on three occasions during the rehabilitation. The results of the hematological and biochemical analyses are presented in Table 3. The fecal analysis showed no evidence of parasitic infestation. The fecal cytologic analysis showed the presence of epithelial cells, a scarcity of white blood cells, the presence of cellular debris, and regular amounts of bacteria. The presence of Gram-negative bacillus and *E. coli* growth was detected in the fecal bacteriological analysis. The bacteriological analysis of the samples taken from the epithelial lesions showed the presence of mixed flora, Gram-positive and -negative cocci, *Staphylococcus* spp., and *Escherichia coli* sensitive to ampicillin and cephalosporins, but resistant to sulfonamides. There was no yeast contamination. The therapeutic approach for the epithelial wounds was based on cleaning the surface with a diluted povidone–iodine solution (Pervinox, Laboratorio Elea, Buenos Aires, Argentina). Once the povidone–iodine dried on the wounds (20 to 30 s), a povidone–iodine cream was applied with an oleo-calcareous liniment supplemented with lanolin. This procedure was performed daily. Additionally, it was treated with cefuroxime 25 mg/kg, BID, for 2 weeks.

### 3.2. Feeding

**Figure 5:**
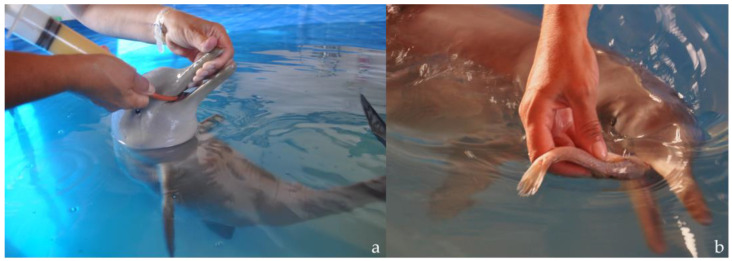
*Pontoporia blainvillei* calf being fed using a feeding tube (**a**) and solid food (**b**) during rehabilitation.

During the first week, only the milk replacer was offered. As the calf refused bottle feeding, this milk substitute was administered every 3 h via a gastric tube (Figure 5a). The daily metabolizable energy (ME) provided by this formula was 938 Kcal, approximately 95.5 Kcal ME/kg; a daily weight gain of 110 g (range 60–240 g) was recorded. The milk substitute was well tolerated, and a positive behavioral response to the feeding tube (swimming directly toward the technician) was observed.

During the second week, the total intake was increased to 1150 Kcal ME/day (approximately 114.16 Kcal ME/kg). Solid foods were introduced and were well tolerated. The formula represented 64%, and the solid food 36%, of the daily diet. The daily weight gain with this formulation was approximately 140 g (range 70–200 g).

During the next period, weeks three to nine, the blended formulation was decreased from 450 g to 200 g, and the solid food increased from 950 g to 1400 g. By week nine, the formula represented 20%, and the solid food 80%, of the diet. The amount of dextrose and fish oil was also gradually reduced from 24 and 6 g to 12 and 4 g, respectively. During this period, the daily caloric intake was about 1750 Kcal ME/day (approximately 119 Kcal ME/kg). At the beginning of this period, the calf’s weight was 10.50 Kg and by week nine it was 13.50 Kg, representing a daily weight gain of about 70 g.

During weeks 10 to 13, the diet provided 1300 Kcal ME/day (about 96.30 Kcal ME/kg). The progressive transition to a completely solid diet was achieved by week 11; after that, the nutritional intake entirely consisted of solid food, (Figure 5b) plus dextrose (10 mL) and fish oil (9 mL).

**Figure 6:**
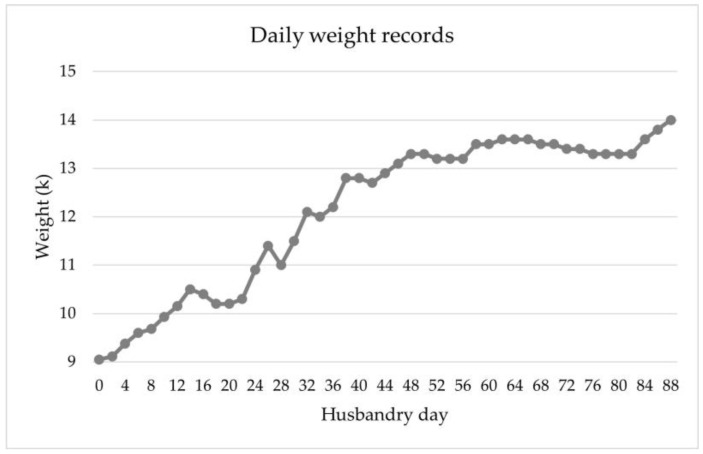
Daily weight of *Pontoporia blainvillei* during 88 days of rehabilitation.

The daily weight gain fluctuated between 0 and 300 g, with an average of 70 g per day. Its daily weight was nearly stable during the first two weeks of this period (weeks 10 and 11; 13.50 ± 0.10 kg). A slight decrease in body weight was observed during the next eight days (13.30 kg), followed by an increase in the following days of up to 14.00 kg. Figure 6 shows the daily recorded weight during the rehabilitation.

The dolphin remained under human care for 88 days, from 23 January to its death on 20 April. The postmortem examination revealed that the lungs exhibited an increased consistency with a dark-red color and multiple small, irregularly shaped, pale-yellow foci, compatible with pneumonia.

## 4. Discussion

The rescued franciscana was a female at the beginning of the weaning period [5]. The initial clinical assessment showed a body temperature of 36.6 °C, which was within the physiologic range, 36–38 °C, reported for this species [6]. However, a lung problem, possibly due to the stranding, was also detected. Following the recommendations of the stranding guide, she was treated with antibiotics and diuretics and supported with a harness [15].

The water quality was maintained as recommended for cetaceans under human care [19], and recently for franciscanas [11,20]. The water salinity of the rehabilitation pool was around 23 ppt, slightly lower than the recent recommendations for stranded franciscanas (25–35 ppt) [6], but within the range reported by Meegan et al. 2022 for the rehabilitation of neonatal franciscana (20–35 ppt) [11]. In the current study, the water salinity was determined based on the average estuarine salinity in the species’ range, and our previous records of an adult franciscana rehabilitated at the FMMRC [7]. Due to the lack of data on thermoneutrality in franciscana dolphins, information from a previously rehabilitated adult specimen and data about their natural habitat, the Río de la Plata estuary [21], were used to estimate the optimal water temperature for thermoneutrality in the current case. Further studies are needed to determine the lower and upper critical temperature values to improve rehabilitation outcomes for this species.

As usually recommended for stranded animals, several samples were taken to assess any clinical conditions. Respiratory tract samples were scarce, and no conclusive data could be obtained from those analyses, even though they were performed several times. In recent years, an endoscopy of the respiratory tract has begun to be used in cetaceans, a technique that has allowed for an evaluation and sampling, with proven utility, of bottlenose dolphins (*Tursiops truncatus*) [22,23,24] and false killer whales (*Pseudorca crassidens*) [22]. Nevertheless, the franciscana dolphin is one of the smallest cetaceans, and there are still significant gaps in our knowledge regarding its physiology and the metabolism of sedative drugs. Moreover, in 2012, the number of comprehensive anatomical studies of the respiratory tract was inadequate to ensure the safety of the procedure. Therefore, exhalation sampling, in conjunction with ultrasound and chest radiography, represented the optimal methodology for the evaluation and diagnosis of the respiratory tract.

The medical treatment used in this case followed the standard recommendations for stranded cetaceans and the previous records on rehabilitated franciscanas at the FMMRC.

Most of the hematological values in the present case are consistent with the previous findings for an adult specimen rehabilitated at the FMMRC [7]. However, in this case, several enzymes were higher compared to the adult franciscana, such as lactate dehydrogenase (510.5 ± 92.63 vs. 243.2 ± 89.9 UI/L, respectively), maybe due to the recovery process; alkaline phosphatase was also higher than in the adult (607.5 ± 7.78 vs. 208.89 ± 149.2 UI/L, respectively), and is usually associated with development; and creatine kinase (235.5 ± 13.44 vs. 41.24 ± 9.2 UI/L, respectively), probably due to tissue damage during stranding. The triglycerides, but not cholesterol, were found to be lower than in the adult (126.25 ± 65.4 mg/%, respectively), probably due to the diet. Although these comparisons were made using samples (*n* = 13) from a single adult animal, to our knowledge, they represent the only reports for the species. The erythrocytes, hemoglobin, hematocrit, and albumin were similar to reports for the common dolphin (*Delphinus delphis*; 5.01–6.42 10^6^/mm^3^, 16.7–19.6 g/dL, 23.80–55.13%, and 3.1–4.3 g/%, respectively) [25].

Due to the lack of information on the nutritional characteristics of the prey of the franciscana and the changes in its dietary intake during the different stages of growth, and because the only milk substitute that is recommended for whales and dolphins (Zoologic^®^ Milk Matrix 30/55, Hampshire, IL, USA) was not available in Argentina [17], homemade formulas were used, and were adapted to the category of animal and body weight gain. The diet formulas were then based on studies of franciscanas in the region, such as their milk composition [18], feeding ecology [8], and previous records from the FMMRC. Subsequent reports on the diet composition and prey size [26], and the calf chronology [5], of franciscana in Argentine waters also support our formulas. With the formulas used in the current case, we recorded a daily weight gain of about 100 g, with an average of 1200 kcal ME/day, similar to the weight gain expected for small species, such as *Stenella* spp., *Delphinus* spp. [17], and young franciscanas [6,11].

Although the animal was not released because it did not reach the live-capture stage during rehabilitation, the dietary approach used during rehabilitation was effective at achieving an acceptable daily weight gain, and the medical treatment was successful at resolving the primary conditions. The Total Energy Requirement (TER) consumed by the growing franciscana was 1.5–2 times the Resting Energy Requirements (RER) calculated [27], similar to that for other carnivores [28]. Although the animal could not be released, this case demonstrates that franciscana calves are promising candidates for successful rehabilitation, unlike newborns, who often arrive in a compromised condition. While each case is unique and presents different challenges, each provides valuable lessons on how to manage these situations. It is only a matter of time before the first successful rehabilitation takes place.

## 5. Conclusions

Although the results are based on a single rehabilitated individual, they represent the first contributions to the knowledge of the rehabilitation and nutritional needs of an individual during the weaning period. These findings provide an important baseline for future studies that could enhance the ability of rescue and rehabilitation professionals and technicians to provide optimal care for debilitated live-stranded dolphins. The medical treatment followed the standard recommendations and previous records for franciscana rehabilitated at the FMMRC. The water quality and salinity were maintained as recommended, providing an optimal environment for the dolphin’s recovery. Despite the lack of conclusive respiratory data, alternative diagnostic methods, such as exhalation sampling, ultrasound, and chest radiography, proved useful. The hematological values in this case were broadly consistent with those of previous findings, with some variations likely due to the recovery process and developmental stage.

The nutritional approach, although limited by the unavailability of the recommended milk substitutes, successfully supported the dolphin’s weight gain and overall health. This formulation could be an alternative for rehabilitation centers without access to commercial milk substitutes. Although the animal could not be released, this case highlights the potential for the successful rehabilitation of franciscana calves and the importance of further research and the adaptation of rehabilitation protocols. Each case provides invaluable insights and brings us closer to achieving successful rehabilitation outcomes for this species. This information may also be useful in the implementation and development of health protocols for this species.

## Figures and Tables

**Table 1:** Nutritional profile, on Dry Matter basis, of different formulas used during franciscana rehabilitation.

Nutrient (Dry Matter)	Rehabilitation Period (Weeks)			
	First	Second	3rd to 9th	10th to 13th
Carnivorous ME (kcal/g)	4.02	4.17	4.16	4.04
Crude Protein (%)	50	55	54	58
Crude Fat (%)	10	12	12	11
Crude Fiber (%)	0.26	0.23	0.23	0.25
Water-Soluble Carbohydrates (%)	25.5	20	20	16
Ash (%)	4.92	4.7	4.6	4.92
Calcium (%)	1.54	1.4	1.4	1.6
Phosphorus (%)	0.6	0.67	0.66	0.7

ME: metabolizable energy.

**Table 2:** The composition of the feeding formula offered to the *Pontoporia blainvillei* during rehabilitation.

	Rehabilitation Period (Weeks)			
Components	First	Second	3rd to 9th	10th to 13th
Hydration salts (Electrolitos Plus, Ruminal, Argentina; mL)	130.00	130.00	130.00	130.00
Shrimp (*Artemisia longinaris*)	153.00	193.50	104.50	59.00
Saracca loin (*Brevoortia aurea*)	148.00	193.50	104.50	59.00
King weakfish (*Macrodon ancylodon*)	209.50	193.50	104.50	59.00
Fish oil (Omega 3, Natufarma, Argentina)	7.50	10.00	5.80	2.20
Dextrose 100%	42.50	32.00	18.00	7.00

Feeding formula was prepared daily. Components are presented in grams per day.

**Table 3:** Hematological and biochemical values of franciscana dolphin (*Pontoporia blainvillei*) calf during rehabilitation.

	Blood Sample Collection During Rehabilitation			Reference
	Day 2	Day 11	Day 23	Values (*n* = 13)
Erythrocytes (millions/mm^3^)	5.21	5.03	6.18	4.60 ± 0.28
Hemoglobin (g/%)	17.70	16.90	17.50	15.64 ± 1.2
Mean Corpuscular Hemoglobin (pg)	33.90	33.50	28.30	33.82 ± 2.3
Mean Corpuscular Hemoglobin Concentration (g/dL)	37.60	37.50	35.00	35.35 ± 2.2
Mean Corpuscular Volume (µm^3^)	90	89	81	95.30 ± 0.2
Hematocrit (%)	47	45	50	44.3 ± 2.58
Platelets (mil/mm^3^)	216	209	332	310 ± 90
Leukocytes (mm^3^)	4000	6400	5200	5908.33 ± 1485
Relative Formula				
Neutrophils (%)	53	58	63	46.17 ± 11.82
Eosinophils (%)	2	2	4	13.17 ± 4.9
Basophils (%)	0	0	0	0
Lymphocytes (%)	40	36	26	38.83 ± 12.8
Monocytes (%)	5	4	7	1.83 ± 1.4
Absolute Count				
Neutrophils	2120	3712	3276	3076.67 ± 885.1
Eosinophils	80	128	208	897.42 ± 466.8
Basophils	0	0	0	0
Lymphocytes	1600	2304	1352	2565.50 ± 844.1
Monocytes	200	256	364	118.75 ± 95.5
Creatinine Kinase (UI/L)		226	245	41.24 ± 9.2
Aspartate Transaminase (UI/L)		24	4	51.54 ± 10.9
Alanine Transaminase (UI/L)		18	4	4.60 ± 1.1
Alkaline Phosphatase (UI/L)		613	602	208.89 ± 149.2
Lactate Dehydrogenase (UI/L)		576	445	243.21 ± 89.7
Gamma GT (UI/L)			34	8.43 ± 2.1
Triglycerides (mg/%)			49	126.25 ± 65.4
Cholesterol (mg/%)			257	281.67 ± 91.6
Creatinine (mg/%)		0.49	0.41	0.76 ± 0.3
Urea (mg/%)		126	110	111.00 ± 5.4
Glucose (mg/%)		68	61	74.08 ± 13.6
Acid Uric (mg/%)			1.60	<2
Proteins Total (g/%)		5.08	6.00	6.71 ± 0.3
Albumin (g/%)		3.18	3.71	
Globulin (g/%)		1.90	2.31	
A/G Relation		1.67	1.61	
Calcium (mg/%)			10.90	9.59 ± 0.3
Phosphorus (mg/%)			5.67	6.89 ± 1.6
Bilirubin Total (mg/%)		0.25	0.29	0.57 ± 0.4
Direct Bilirubin (mg/%)		0.03	0.03	
Indirect Bilirubin (mg/%)		0.22	0.26	
Amylase (U/L)			126	572.83 ± 40.7
Sodium (Mmol/L)			158	151.29 ± 3.2
Potassium (Mmol/L)			4.20	4.79 ± 0.2

Reference values (mean ± SD) correspond to records of *n* = 13 blood sample analyses from one adult *Pontoporia blainvilei* dolphin rehabilitated at FMMRC between 1996 and 1999.

## Data Availability

The data presented in this study are available on request from the corresponding author.
